# Tracking Resilience to Infections by Mapping Disease Space

**DOI:** 10.1371/journal.pbio.1002436

**Published:** 2016-04-18

**Authors:** Brenda Y. Torres, Jose Henrique M. Oliveira, Ann Thomas Tate, Poonam Rath, Katherine Cumnock, David S. Schneider

**Affiliations:** 1 Program in Immunology, Stanford University, Stanford, California, United States of America; 2 Department of Microbiology and Immunology, Stanford University, Stanford, California, United States of America; 3 Department of Biology and Biochemistry, University of Houston, Houston, Texas, United States of America; Princeton University, UNITED STATES

## Abstract

Infected hosts differ in their responses to pathogens; some hosts are resilient and recover their original health, whereas others follow a divergent path and die. To quantitate these differences, we propose mapping the routes infected individuals take through “disease space.” We find that when plotting physiological parameters against each other, many pairs have hysteretic relationships that identify the current location of the host and predict the future route of the infection. These maps can readily be constructed from experimental longitudinal data, and we provide two methods to generate the maps from the cross-sectional data that is commonly gathered in field trials. We hypothesize that resilient hosts tend to take small loops through disease space, whereas nonresilient individuals take large loops. We support this hypothesis with experimental data in mice infected with *Plasmodium chabaudi*, finding that dying mice trace a large arc in red blood cells (RBCs) by reticulocyte space as compared to surviving mice. We find that human malaria patients who are heterozygous for sickle cell hemoglobin occupy a small area of RBCs by reticulocyte space, suggesting this approach can be used to distinguish resilience in human populations. This technique should be broadly useful in describing the in-host dynamics of infections in both model hosts and patients at both population and individual levels.

## Abstract 2

This study shows that infections cause sick hosts to loop through disease space on their return back to health; resilient individuals take tiny loops through this space, minimizing the impact of the infection.

## Author Summary

When we get sick, we long for recovery; thus, a major goal of medicine is to promote resilience—the ability of a host to return to its original health following an infection. While in the laboratory we can study the response to infection with precise knowledge of inoculation time and dose, sick patients in the clinic do not have this information. This creates a problem because we can’t easily differentiate between patients who are early in the stages of infection that will develop severe disease from more disease-tolerant patients who present later in the infection. The distinction between these two types of patients is important, as the less disease-tolerant patient would require a more aggressive treatment regime. To determine where patients lie along the infection timeline, we charted “disease maps” that trace a patient’s route through “disease space.” We select symptoms that produce looping graphs as patients grow sick and recover. Using a mouse–malaria model, we demonstrate that less resilient individuals take wider loops through this space, representing a longer infection time with more severe symptoms. We find this looping behavior also applies to humans and suggest that people carrying the sickle cell trait are more resilient to malaria infections.

## Introduction

As a field, we study infectious diseases to learn how to reduce their impact. One common method is to use antibiotics to limit pathogen growth. An alternative approach, which potentially avoids the risk of pathogen evolution of antibiotic resistance, is to limit the damage caused by the infection [1]. To study infection-induced pathogenesis, we need reliable methods of quantifying its measurement. One method that has been used to study the pathogenesis of infectious diseases in populations is to measure disease tolerance [2–5]. Disease tolerance is a dose response curve that summarizes the entire infection by plotting summary health and microbe measurements for each infected individual. For example, mouse strains can be differentiated with respect to their disease tolerance to malaria by plotting graphs correlating the lowest red blood cell count (minimum health) by maximum parasite density and demonstrating that the curves have different slopes [1]. When enough data is gathered from a population, this produces a disease tolerance curve, and the shape of that curve provides insight into the disease; for example, if we learn that the curve is sigmoid, we identify three to four parameters (baseline, sensitivity, maximum, and slope) that we can alter to manipulate the health of the host [6].

A frustration with this sort of disease tolerance analysis is that the method works in model systems but can’t be applied to patients suffering from acute infections. There are two barriers that limit the usefulness of disease tolerance in humans: First, we cannot ethically gather longitudinal data from most infected patients, and longitudinal data is required for the summary measurements described above. The reason for this is that we need to treat an acutely sick patient when they present at the clinic and cannot wait for them to reach their maximum parasite load and minimum health before offering treatment. Second, disease tolerance is a property of populations and not individuals [7]. Even if we could gather the necessary longitudinal data from a patient, the data wouldn’t define disease tolerance for that patient; we would obtain a single datum that we could place on a health by microbe plot, but we would not know what curve we should draw through that point. If we stick to formal definitions of disease tolerance as requiring stereotyped summary data, we will be unable to apply it to the system in which we would like to use it most.

We sought alternate methods for visualizing and quantitating the relationships between health, microbes, and the immune response using the sort of data we can gather from patients. We focused on resilient individuals who get sick and then recover to their original state, because our ultimate goal is to take sick patients and nudge their path toward resilience. We imagined a multidimensional space that can be plotted using quantitative measurements of disease symptoms as axes [8,9]; we could use this space to follow the path patients followed as they grew sick and then recovered. Instead of mapping summary measurements and finding the shape of that curve, we define the curves traced by infected individuals as they travel through this “disease space.” We are particularly interested in hysteretic relationships, where by “hysteresis” we mean that the current conditions are defined by a memory of past events and aren’t simply a response to the immediate environment. The result of hysteresis during an infection is that the route an infected host takes to maximum microbe load in microbe by immune response space differs from the route back to health. One simple way that hysteresis can be generated is if there is a time lag between an event and the outcome, because it takes time to synthesize a product; in this case, the outcome will show hysteresis with respect to the inducer. If the shape of the path a resilient patient takes through disease space is a loop, then we should be able to monitor variations in resilience by observing changes in the shape of these loops.

Here, we show that such looping curves are a common motif in a model malaria infection in mice. We use these curves to define “disease maps” that plot the route individuals take through disease space as they sicken and recover or die. We developed methods for visualizing this route in cross-sectional data using nearest neighbor and topological data analysis. Once we demonstrated that we could identify and reconstruct these loops from cross-sectional data, we developed a method of measuring deviations from the resilient path. We considered the data to trace circles in disease space and transformed the data from Cartesian to polar coordinates. This enables us to identify dangerous neighborhoods of disease space using probabilistic statistics. This approach has the benefit of suggesting an order for points in a cross-sectional analysis that lets us hypothesize a disease path for an infection.

## Results

### Identifying Looping Curves in Disease Space

**Fig 1:**
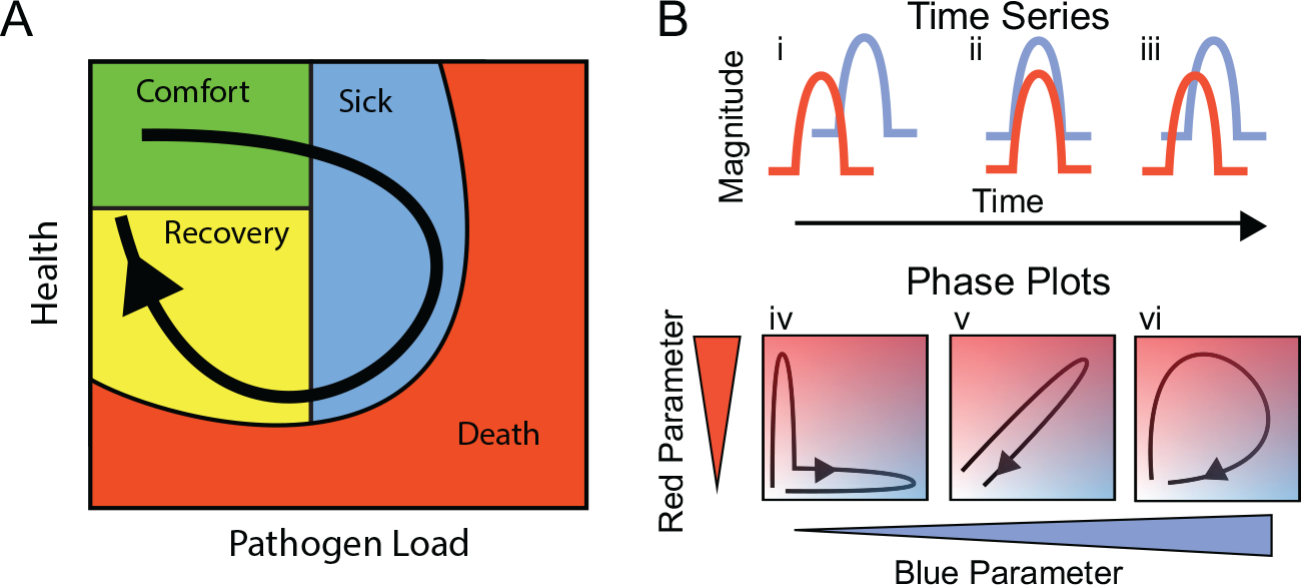
Predictions of disease space map behavior. (A) This figure imagines a map tracing the path that a resilient infected individual might travel through disease space. The host starts at a position of comfort, but as pathogen load increases and health decreases, it enters a state of sickness. Once pathogen load decreases, the host starts to actively repair its health, resulting in an open loop in which every position along the path is unique. (B) For a system in which two parameters oscillate through a single cycle, they will trace three basic shapes through phase space. If the parameters do not overlap with each other in time, they will trace an L-shaped curve (i, iv). Complete overlap produces a line (ii, v), and partial overlap produces a loop (iii, vi). The color gradients show increasing concentrations of each parameter.

We started with the proposition that infected patients will trace a path over a multidimensional manifold in disease space; resilient patients will travel predictable paths as they sicken and recover, and patients who do poorly will also take predictable paths when they sicken and die. We can examine a patient’s progress along this disease path by observing two-dimensional projections of this space using readily measured symptoms. We call these two-dimensional shadows of the multidimensional manifold “disease maps.” These maps trace the route a patient follows as they travel from a region of comfort through sickness and back along a path to recovery (Fig 1A). Disease maps have the potential to be useful because they define a patient’s position in disease space, and, with experience, we could predict whether this position places the patient on a well-travelled route back to health or whether they are headed into a dangerous neighborhood where they risk permanent harm or death. We hypothesize that these dangerous neighborhoods lie on the outside of the paths taken by resilient individuals; extending this idea, we expect that resilient individuals will take tight loops through disease space while those at risk of severe pathology or death will take wide loops.

We can predict three basic structures for simple disease maps. If two parameters are completely out of phase with each other, when plotted in phase space they will trace a boomerang-shaped curve that isn’t particularly enlightening (Fig 1Bi and 1Biv). If two parameters are in phase with each other, we can plot the correlation between these and determine parameters like slope, for example, if the two parameters have a linear relationship (Fig 1Bii and 1Bv). We are most interested in cases in which the two parameters are partially out of phase with each other such that they form a hysteretic looping curve like that shown in (Fig 1Biii and 1Bvi). The utility of these curves is that each point along the curve defines a patient’s travel through disease space uniquely, as the patient does not retrace their steps. Thus, we can determine where the patient lies along a disease path, which is something we can’t do with a simple linear correlation.

**Fig 2:**
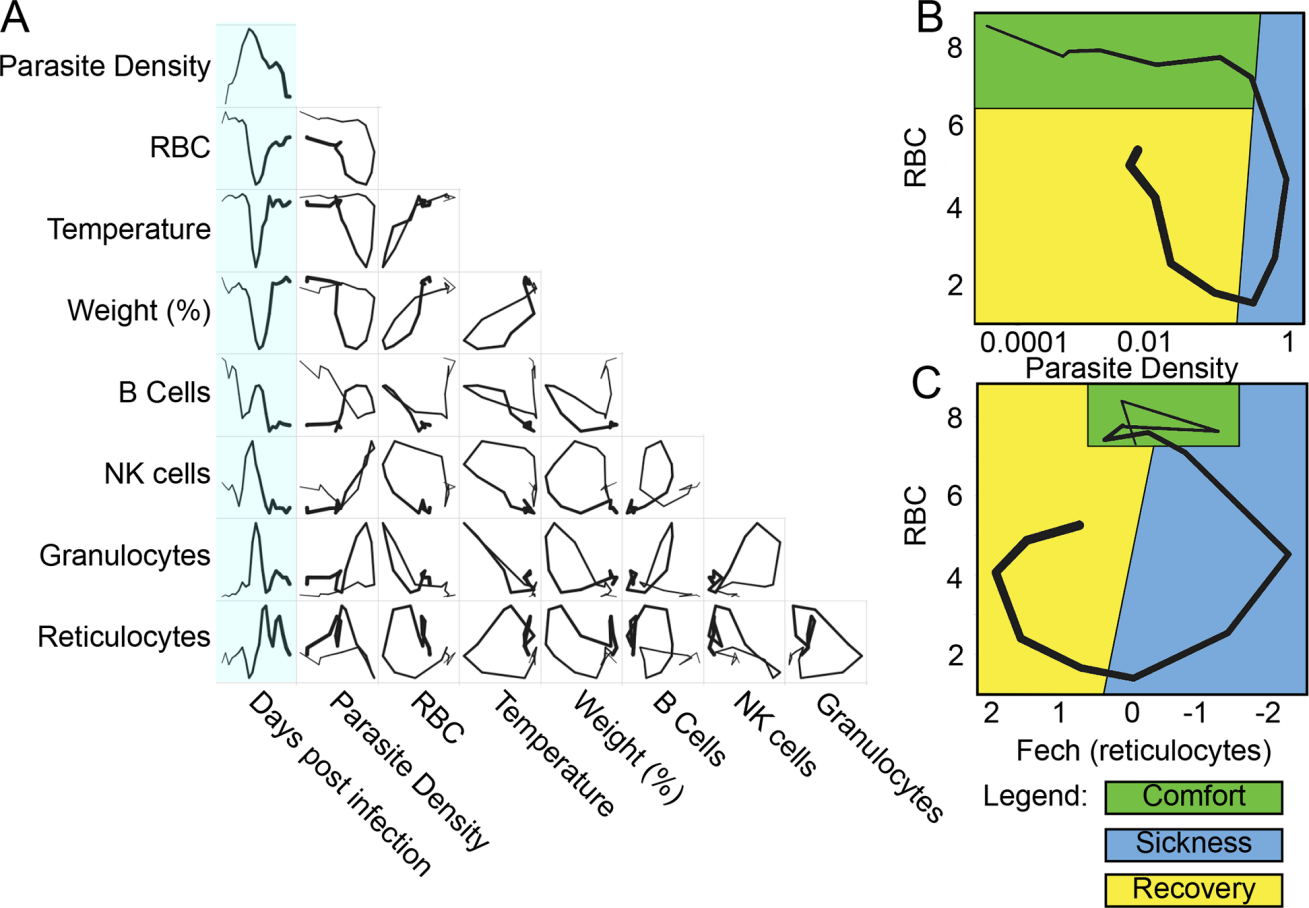
Disease space maps of malaria-infected mice. (A) Average values for eight parameters for three mice measured (and averaged) daily for 20 d are plotted in a timeline marked in blue. The paths for the three mice plotted individually are shown in S2 Fig. The transcript markers used to define B cells, NK cells, granulocytes, and reticulocytes are, respectively, Cd79b, Nkg7, Camp, and Trim 10, which are reported as log_2_ values. Time is indicated by the increasing thickness of the curve. (B,C) Phase plots for representative looping pairs of parameters. Note that the axes have been flipped so that all graphs start at the top, and the sick mice follow a clockwise path through phase space. The graph shows “comfortable” (days 0–6, green), “sick” (days 7–10, blue) and “recovering” (days 11–15, yellow) regions. See also S1 Fig.

To establish which parameters should be plotted to build informative disease maps, we gathered a multi-dimensional dataset over the course of infections of mice challenged with the malaria parasite *Plasmodium chabaudi*. We chose this parasite because it is readily measurable in the blood of the host and causes pathology that can also be measured in the blood; another useful feature is that the parasite causes a self-resolving infection. Together, these properties let us easily follow the progression of an infection in a resilient system. We mapped the behavior of circulating blood cells and parasites by following infected mice over the 26 d infection course and performing microarray analysis on daily blood samples. We grouped the transcriptome data using a k-means analysis, and these groups were characterized as reporting circulating cells based on the composition of their members (S1 Fig, S1–S3 Tables) [10,11]. We plotted pairs of these k-means averages against each other to observe the phase curves and found hysteretic relationships that produced open loops that could be used as maps (Fig 2A). In Fig 2B and 2C, we show how some of these loops can be considered with respect to the cartoon model of disease space we introduced in Fig 1A.

We developed a simple computational method of identifying looping pairs of parameters that depends on the expected geometry of the interaction. We reasoned that if a pair of parameters traced a loop, this would circumscribe a larger area than other types of curves; therefore, we made pairwise comparisons of 2-fold regulated transcripts creating phase curves for each pair and then measured the area enclosed by the curve. This approach computationally identified the same families of curves that we found by plotting the k-means groups and identifying looping curves visually (S4 Table). The genes identified in this analysis for the largest loops came from k-means clusters that were enriched for granulocyte- or reticulocyte-specific gene markers.

### Building Disease Maps with Cross-Sectional Data

An issue with building disease maps for humans is that this approach suffers from the same problem as tolerance curves; we typically cannot obtain the longitudinal data required to trace these routes. When a child arrives at a clinic with uncomplicated malaria, they are tested, treated, and sent home. We expect the resulting cross-sectional results to produce clouds of multidimensional data points, because infected individuals are sampled at different points during the infection. Hysteretic correlations that produce looping paths could easily be misinterpreted using standard methods of fitting mathematical functions to such data, because we tend to fit monotonic functions. For example, faced with a looping set of data like that shown for red blood cells (RBCs) by natural killer (NK) cells, it is easy to imagine how we might attempt a correlation analysis and conclude that there was no correlation between the two parameters because the common linear, exponential, or sigmoid relationships we attempt to fit to our data don’t do a good job of modeling these data.

We sought a method of tracing the path sick individuals travel through disease space using only cross-sectional data. We hypothesized that if each patient sample was plotted in disease space, then a given host would most resemble individuals on a similar path. If we connected each individual to its nearest neighbors in multidimensional space but plotted the data in a lower-dimensional space, we could trace the disease map taken by the sick hosts. We used two approaches to make disease maps from cross-sectional data: nearest neighbor analysis and topological data analysis.

**Fig 3:**
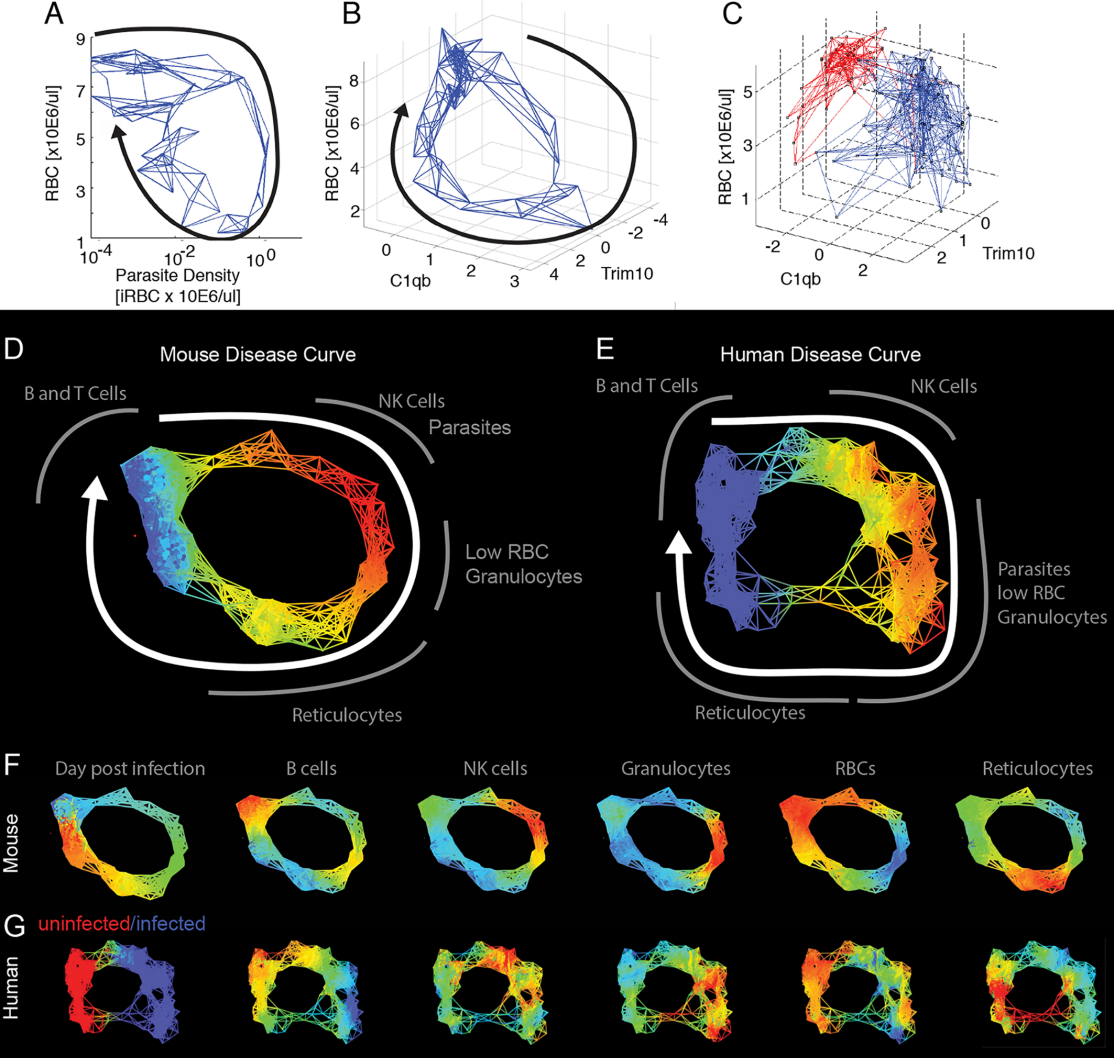
Reconstructed mouse and human disease space maps from longitudinal and cross-sectional data. (A–C) show nearest neighbor networks for mouse and human data plotted in physical spaces. Arrows indicate the direction of movement through phase space. (A–B) show longitudinal data. (C) shows cross-sectional data. (A) Mouse red blood cell (RBC) by parasite density plotted by connecting five nearest neighbors. (B) Mouse RBC by C1qB by reticulocyte (Trim10) connecting five nearest neighbors. (C) Human RBC by C1qb by Trim10 map connecting five nearest neighbors. Children reported as “uninfected” are marked in red, while those infected are marked in blue. Topological network maps of mice (D) and humans (E) suffering from malaria. The known timeline in the mice runs clockwise as marked by the white arrow. The inferred human timeline is marked similarly. The color scheme in (D) and (E) marks parasite density, where blue represents low values while red represents high values. Segments of the map are marked in grey to show transcript or cell counts reporting the relative abundance of marked cell types. Red blood cells were counted directly using flow cytometry. The markers for B cells, granulocytes, NK cells, and reticulocytes are: Faim3, Lcn2, Nkg7, Trim10 for both mice and humans. (F,G) show mouse and human malaria maps reporting different parameters. Colors mark the progression of time or the relative abundance of marked parameters. Ranges for (D–G) and parameters for deriving the graphs are listed in S6 Table.

To examine the data using nearest neighbor analysis, we took the 78 time points from the study in which we followed three mice longitudinally and stripped these data of time information. We then plotted the remaining data in two- and three-dimensional spaces that we had identified as producing looping hysteretic curves. We then connected individual data points to their five nearest neighbors using a subset of the transcriptome data that focused on identifiable cell types (S5 Table). We chose five nearest neighbors as, with this data set, this number produced a graph that revealed the shape of the disease path without being overly dense. This generates a network, and the shape of that network overlapped with the actual paths the mice took through disease space (Fig 3A and 3B). We extended this approach to published data for humans, analyzing cross-sectional transcriptome results from the blood of malaria-infected and uninfected children (Fig 3C) [12]. Comparison with the mouse disease map suggests that the human infection also follows a loop, though the loop has an obvious low-density gap corresponding to the recovery stages of the disease.

We applied topological data analysis (TDA) techniques that cluster the data without imposing a connection structure such as a hierarchical pattern or least branching tree [13]. This creates non-dimensional networks in which it is easy to visualize the underlying shape of the data and to compare graphs between organisms. The topological networks provide a striking representation of the health space that resembles the disease maps imagined in Fig 1A, in which distinct regions of the networks correspond to distinct parts of the disease: comfort, sickness, and recovery. Both the mouse and human datasets form looping structures (Fig 3D–3G, S5 and S6 Tables). By mapping the intensity of parameters such as parasites, RBCs, granulocytes, or reticulocytes, it becomes clear that the mouse and human infections are collinear in many respects, having the same order of events (Fig 3F and 3G). As was seen in the nearest neighbor analyses above, the graph of the human data suggests that the children in the “uninfected” group are not homogeneous. One inference we gain from this analysis is that a fraction of these “control” children may be recovering from malaria infections but had parasite loads below the limit of detection. This is suggested because of the relatively high reticulocyte and low granulocyte levels seen in recovering mice is also seen in children in the “uninfected” lower left-hand quadrants of the graphs (Fig 3C).

### Statistical Analysis of Deviations from a Resilient Disease Path

**Fig 4:**
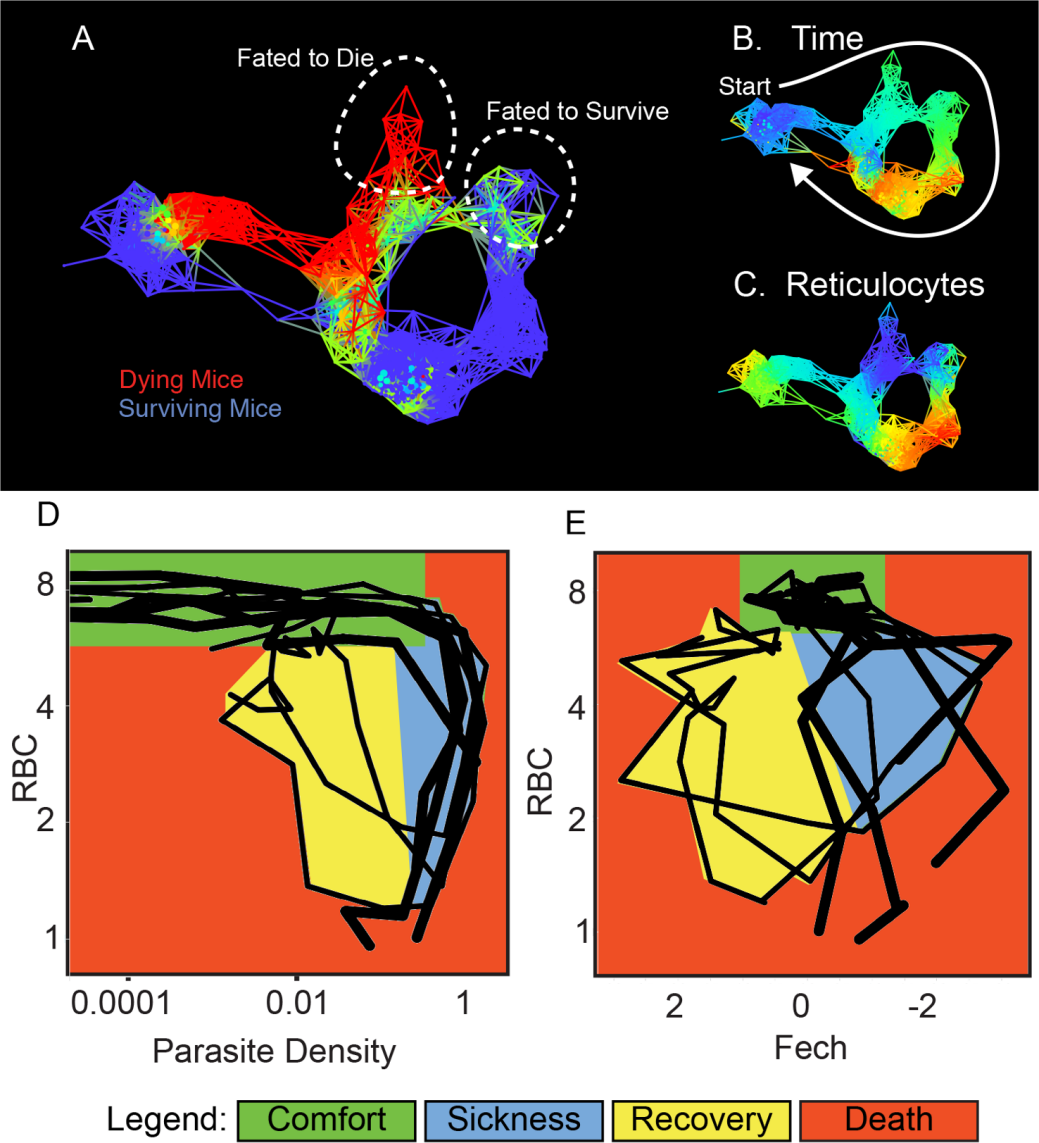
Disease maps of mice with warped disease spaces. (A) A topological network map for malaria-infected mice following the mice for a maximum of 26 d post infection. The surviving mice are marked in blue (*n* = 3), while those who died are marked in red (*n* = 4); other colors show overlap in the map. (B–C) show the same disease map as in (A), but colored according to (B) time or (C) reticulocytes (Ferrochelatase). Phase plots for parameters parasite density by RBC (D) and Fech by RBC (E) that deviate in looping systems in dying mice. Note that the axes have been arranged (D–E) so that all graphs start at the top left and the sick mice follow a clockwise path through phase space. The graph shows “comfortable” (days 0–6, green), “sick” (days 7–10, blue), and “recovering” (days 11–15, yellow) regions. Areas not encompassed by the paths followed by surviving mice are colored red and reveal the dangerous spaces traversed by dying mice. The path of dying mice is outlined in thick lines compared to the thin lines used for survivors. Ranges for (A–C) and parameters for deriving the graphs are listed in S8 Table.

The *P*. *chabaudi* strain we used to infect mice produced 20% lethality; we used TDA analysis to make a graph that separated the living and dying mice into two different paths and then determined how gene expression differed between the two groups (Fig 4A–4C, S7–S9 Tables). This analysis demonstrated that RBCs and reticulocytes differed in their representation in living and dying mice as their paths through disease space separated. This suggested that RBC by reticulocyte graphs could provide a useful disease space for differentiating living and dying mice, unlike the RBC by parasite density space (Fig 4D and 4E). Reticulocytes are RBC precursors, and it makes biological sense that this space would be revealing, as anemia is a major source of pathology in these infections. If reticulocyte production is out of phase with RBC depletion, this could lead to a state in which RBCs dropped to lethal levels before they could be replaced.

**Fig 5:**
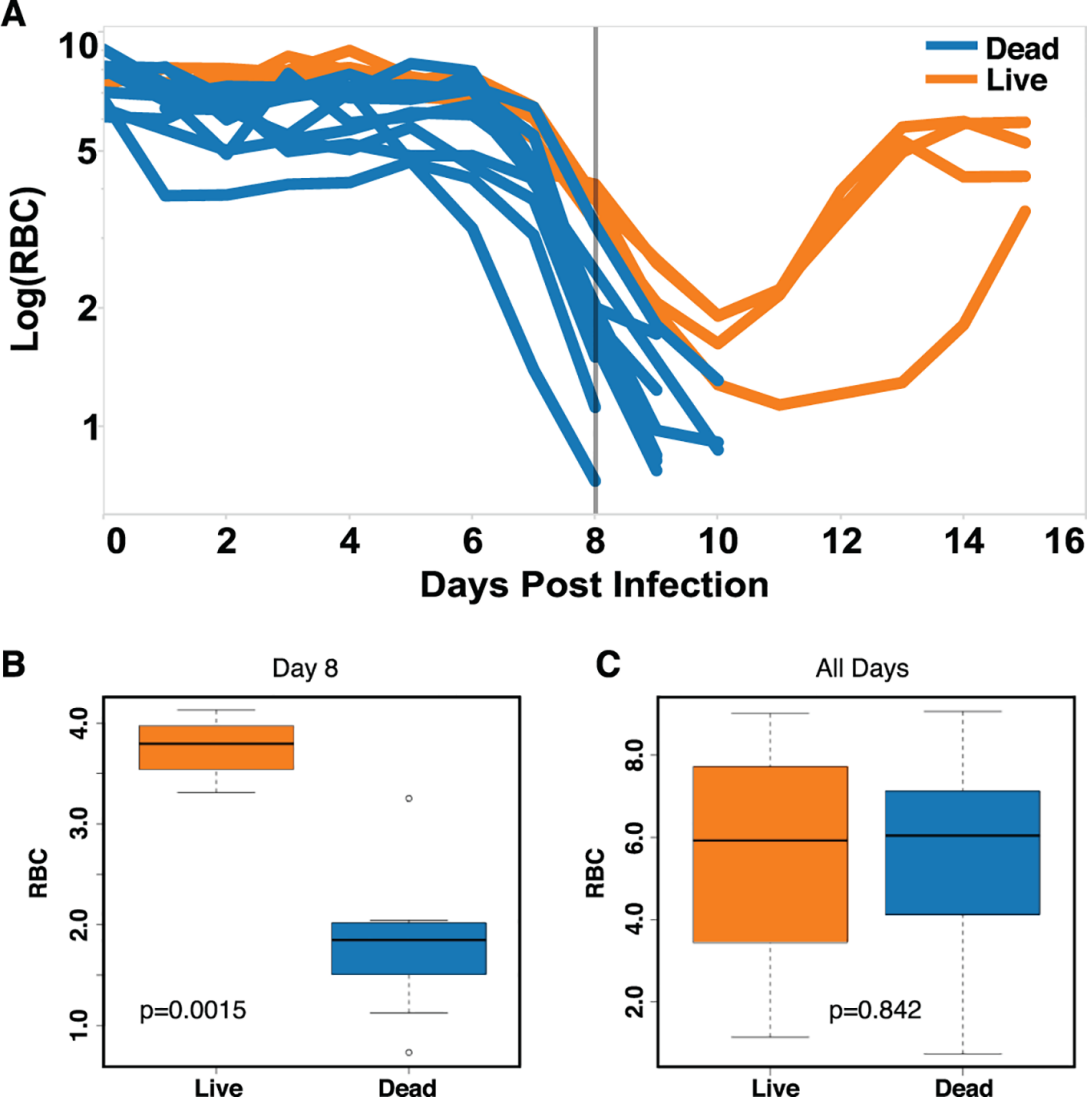
Prediction of mice fated to die based on anemia. (A) Time series data of RBCs for survivors (*n* = 4, orange) and non-survivors (*n* = 11, blue). (B–C) Box plots of RBC counts on day 8 (B) and all days (C) of the infection. Significant difference between conditions on day 8 *p*-value = 0.0015. The *p*-value when considering all of the time points was *p* = 0.842. Data provided in S1 Data.

It would be useful to measure deviations in the path sick individuals took through disease space using a small number of parameters, like RBC and reticulocyte counts, that could be gathered in a physician’s office rather than a full microarray or flow cytometry analysis of the blood. If we plot our mouse data in a time series (Fig 5A), it is easy to see that the mice that are fated to die become anemic earlier than the resilient mice; thus, a single parameter could be used to predict the fate of these experimental mice (Fig 5B). We can do this in the laboratory because we know when we infected the mice, but we can’t expect a child suffering from malaria to tell us when they were bitten by an infected mosquito; therefore, we can’t depend on a time series for diagnosis in a real medical situation. It is going to be rare that we can ever precisely define time zero for infection in the field. To illustrate this point, we find that if we don’t use time post infection in our analysis of the mouse data and consider all of the data points at once, as we would have to with cross-sectional data, we find no predictive value of RBC levels in our mouse analysis (Fig 5C).

**Fig 6:**
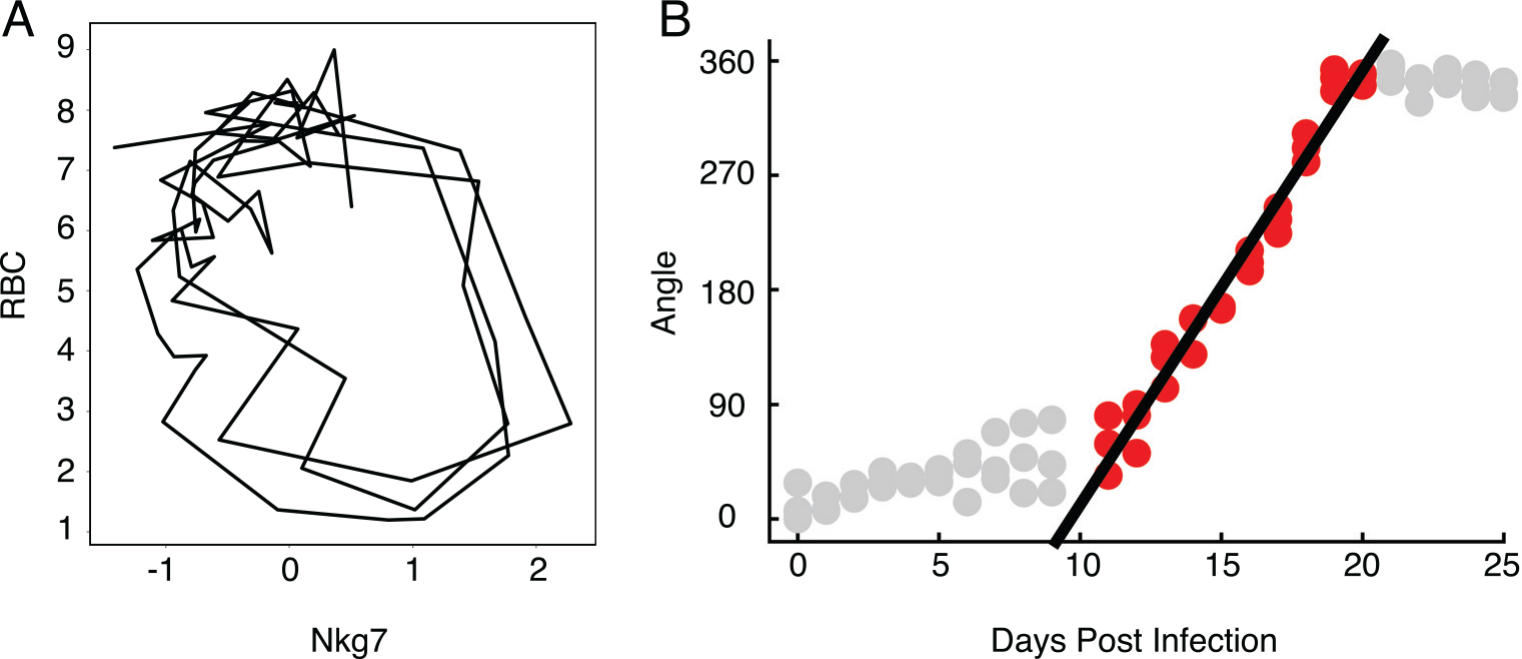
The relationship between time and angle in a disease map. (A) Disease map of live mice (*n* = 3) through Nkg7 by RBC space. (B) Linear correlation between angle and days post infection from day 11 to day 20 (r^2^ = 0.942). Only points colored red were included in the regression analysis. Data provided in S2 Data.

We reasoned that it might be possible to discover the order of events in an infection using a looping disease curve because each point has a unique position along the loop. This would allow us to compare individuals at similar segments of the infection rather than consider the entire course of infection. The path mice take through RBCs by NKG7 space is a useful space to use to describe this process, as the disease curve traced by mice in this space is nearly circular (Fig 6A). Instead of recording these data points in terms of their (*x*,*y*) position in space, we transformed them to polar coordinates. This reports each point in terms of the distance from the center of the loop and their angle from an arbitrary origin that we positioned at the start of the infection; the angle provides a measure of how far the host has progressed along the infection path. If we plot angle versus time, we find a linear correlation (r^2^ > 0.96) over much of the curve (Fig 6B), demonstrating that we can recover the order of events from cross-sectional data using this polar transformation approach; thus, looping disease curves can serve as clocks that report the time-independent order of events in a disease.

**Fig 7:**
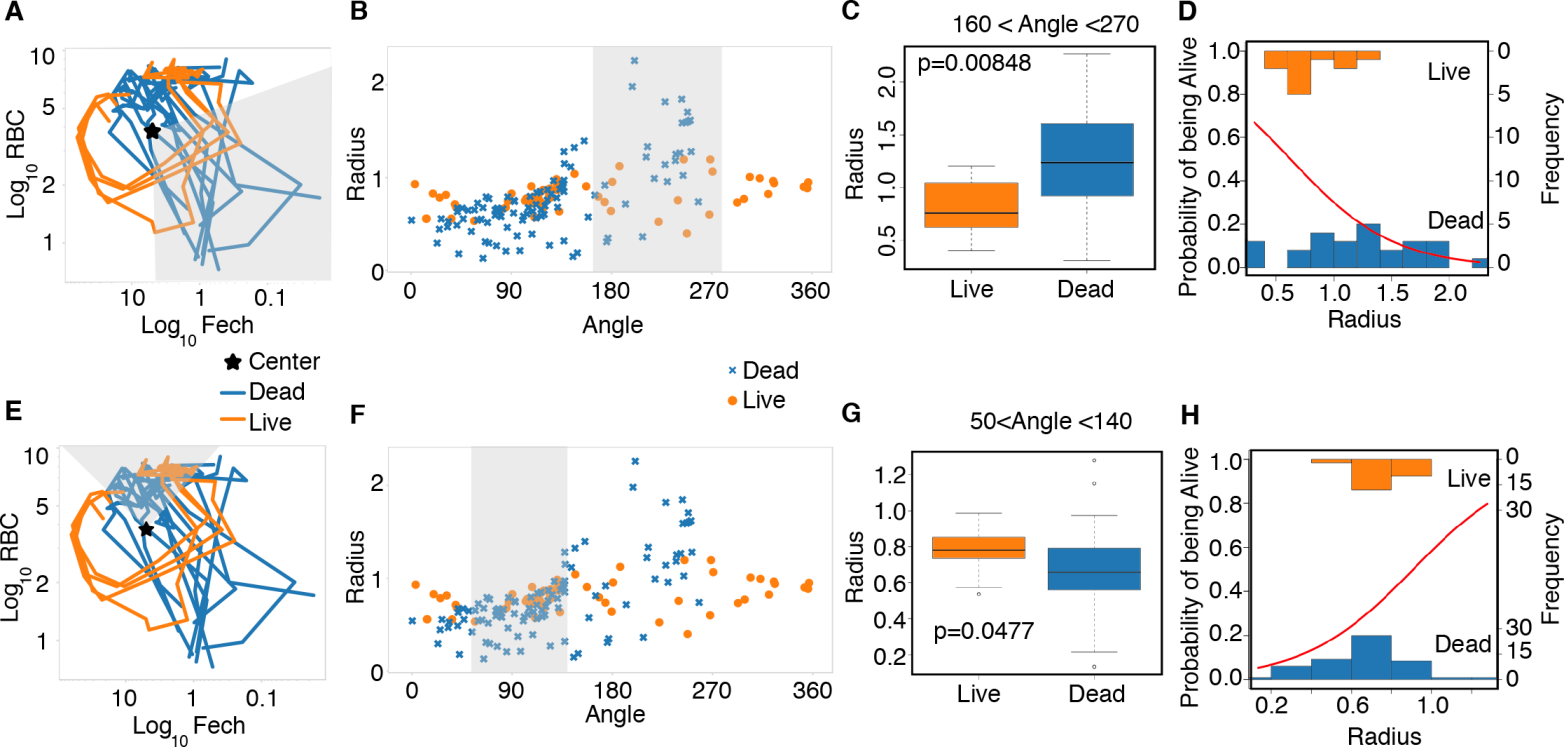
Prediction of mice fated to die using polar transformed data. (A) Disease map of live (*n* = 4) and dead (*n* = 11) mice in Fech by RBC space. Data collected through qRT-PCR. The grey area shows the range of angles analyzed just before the time of death. (B) Radius and angle for live (circle, orange) and dead mice (x, blue). (C) Box plot measuring the radius of live and dead mice. (D) Binomial generalized linear model (glm) showing the probability of survival decrease as the length of the radius increases (red line). Observed values are plotted as histograms. (E) Disease map of live (*n* = 4) and dead (*n* = 11) mice in Fech by RBC space. The grey area shows the range of angles analyzed around time zero. (F) Radius and angle for live (circle, orange) and dead (x, blue) mice at early time points. (G) Box plot measuring the radius of live and dead mice at early time points; *p* = 0.0477. (H) Binomial generalized linear model (glm) showing the probability of survival decrease as the length of the radius increases (red line) for early time points. Data provided in S3 Data.

Once cross-sectional data has been ordered using a polar transformation, we can easily analyze deviations from the resilient path at the point of separation. If we plot RBC numbers by reticulocyte numbers predicted from the microarray in Cartesian space, we find that the resilient mice loop and those fated to die explore areas outside of this loop (Fig 7A). This danger zone is difficult to define in a Cartesian plot because we have to follow variation in two dimensions using a small number of samples. To explore this relationship further, we collected a larger dataset from mice that lived or died during the infection (four surviving and 11 dying mice) and tracked RBCs by flow cytometry and reticulocyte counts by quantitative reverse transcription polymerase chain reaction (qRT-PCR) (Fig 7, S10 Table). When we plot radius versus angle (polar transformed), data that formed a circle in Cartesian space are plotted as a line, and data that deviates from the circle rises above or below the line (Fig 7B). We can analyze these data by performing an ANOVA over interesting ranges of the angle. We transformed these RBC by reticulocyte data to polar coordinates and compared the animals over the angles corresponding to the period where the dying mice diverged. We found that the dying mice differed significantly in terms of radius with respect to the surviving mice (Fig 7C and 7D). Further examination of the mice in polar space showed that we could find a significant difference in radius at the start of the infection, suggesting that there were pre-existing conditions in these mice that made them susceptible to death upon infection with *P*. *chabaudi* (Fig 7E–7H).

**Fig 8:**
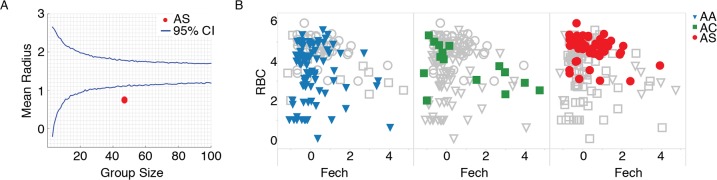
Disease space analysis of malaria-infected children. (A) The mean radius for individuals with the sickle cell trait (AS, red) is below the average random distribution for a group size of 47 patients. (B) Marking individuals with Hemoglobin A (AA, blue, triangle), Hemoglobin C (AC, green, square), and Hemoglobin S (AS, red, circle) in RBC by reticulocyte (Fech) space. Individuals with Hemoglobin S form a smaller cluster than the other two hemoglobins, suggesting a smaller route through disease space. See also S2 Fig. Data provided in S4 Data.

These experiments suggest that if we select a disease space in which sick individuals trace a loop, and that loop is a good indicator of disease, then the most resilient individuals in a population will trace the tightest loops. To test this idea, we examined published genetic variation and transcriptome data from malaria-infected children to determine whether polymorphisms known to limit the severity of malaria restricted patients to a narrow window of disease space [12]. To provide a statistical analysis of these data, we determined the probability that a randomly selected group of data points in this set would produce a cluster of a particular sized radius (Figs 8A and S3). To measure the distribution of small groups of varying sizes, we performed a bootstrap analysis, recording the calculated radii of 1,000 randomly chosen clusters from this dataset, ranging from two to 100 members. This gives us a sense of the distribution the radii would have for given group sizes if the members were chosen randomly. The resulting curve demonstrated that small groups have a relatively high radii variance and that mean radii variance plateaus once group sizes pass approximately 20 members. The dataset used here does not provide enough power to perform a genome-wide association study (GWAS) screen to identify SNPs from the whole genome, but it is powerful enough to let us ask hypothesis-based questions about individual polymorphisms.

Using this approach, we determined whether a polymorphism previously found to associate with malaria defined significantly smaller loops in phase space than might be expected randomly. It has long been known that sickle cell hemoglobin S (AS) reduces the pathology of malaria in patients heterozygous for this allele. We plotted the location of HbS heterozygotes as compared to all other hemoglobin variants and found that the HbS patients clustered together in a relatively small space, near the position of uninfected patients (Fig 8B). The mean of the radii for the cluster of AS patients was smaller than the mean radii for a group of similar size that used the whole dataset; this suggests that small cluster of AS-positive samples was not a random occurrence. This is the pattern we expect for resilient patients.

## Discussion

Once a patient is infected by a pathogen, we have two therapeutic routes to improve their outcome: First, we can reduce pathogen loads through anti-pathogen treatments. Second, we can specifically reduce the accumulating pathology without necessarily altering microbe loads. To accomplish the latter, we need to develop methods of determining when an individual is suffering from unusual pathology and push their physiology back to a less dangerous state. Unfortunately, the methods the field of disease ecology has used to measure these correlations are difficult to gather from patients; the correlations we can make for health and microbe load fall outside of well-behaved linear, logistic, and exponential relationships that are easy to quantitate. Here, we describe a simple pattern that is traced as a resilient individual passes through disease space; these patients loop back to their original positions, and we show that that deviations from these paths can be easily measured using geometrical approaches used to describe circles.

In laboratory experiments, it is possible to identify at-risk animals by following single symptoms using time series analyses, but we can only do this because we know the time post infection. If we know time, we can choose which data points we compare; we don’t have this luxury of aligning data points when dealing with patients, because they walk into a clinic sick and need to be dealt with immediately, and not every patient chooses to go to the clinic at the same stage of the disease. Our work shows that by measuring the correlation between two parameters that are modulated out of phase with each other across the course of an infection, we can create looping maps of disease space. The advantage of a loop is that each point identifies a unique spot on the infection path and can serve as a map of the infection’s progress. These maps define safe neighborhoods that indicate an individual is headed toward recovery and others that suggest an infected individual has a high probability of dying.

It is simple to show these looping maps exist using longitudinal data gathered from *P*. *chabaudi*-infected mice. By correlating gene expression patterns characteristic of circulating cell types to each other and with physiological readouts like weight, temperature, parasite load, and RBC counts, we find that these hysteretic loops are common. To find these relationships in cross-sectional data, we need to develop models that predict the order of patients along the path of disease progression.

The first method we used to predict the order of cross-sectional data was to use a nearest neighbor analysis to define the shape of a correlation between two parameters. We started by plotting data points in a physical space on a graph and drew edges between points and their nearest neighbors as calculated from a high-dimensional space using microarray data. This reconstructs the path individuals take through disease space. When comparing the results from our mouse malaria infection data to published human infection data, we find the mice trace a full loop whereas the humans trace only a portion of a loop. We anticipate this is a fault of the way the data are gathered; patients are admitted into the study when they come to the clinic, and they come to the clinic because they feel ill. Not everyone will choose to go the clinic at the same point in the infection, and there could even be a parental contribution in the determination of when a child is sick enough that they should be brought to the doctor. This will ensure that many data points are gathered for the portion of phase space in which the patients are sick and getting sicker, but recovering patients will be missed because it is wasteful to go to the clinic if you are clearly getting better. The result is that the shape of the correlation could easily be mischaracterized as an arc rather than a loop, because we need to record both pathogenesis and recovery data to define the shape of disease space. Using the same data, topological data analysis produces graphs that are more obviously looping, because the topological networks pull gaps closed. The points in the recovery part of disease space are important for the analysis of resilience. We can imagine what would happen if we gathered data that was distributed across the whole range of the infection rather than using data that focuses on when patients present at the clinic. If we measure the radii of random groups of varying sizes in the Idaghour et al. data [12], we find they have a broader distribution than if we measured points in a set of randomly distributed data covering the same circle (S3 Fig). This suggests it would be possible to increase the power of a resilience analysis by gathering data that was better distributed around the whole disease path.

Once we understand that these data loop, we can use this information to transform the data to a more readily analyzable form by converting it to polar coordinates. This is useful for two reasons: First, this changes the problem of having to account for variance in two dimensions to a simpler situation in which we need to account for just one dimension of variance. For example, this lets us determine that mice with low reticulocytes and RBCs are likely to die when we can’t make that determination using either one of these parameters on their own. Second, this approach can be used to order cross-sectional data along a disease path. This isn’t critical in laboratory studies in which we know the time post infection, but it provides a new tool for studying infected populations. This ordering can be useful because it lets us define a model for changes in a parameter over the course of a disease; in turn, this lets us measure an individual’s deviation from that model.

This idea that resilient patients trace small loops through disease space allows us to explain how some genetic polymorphism could affect the course of a malaria infection. By measuring the radii of infected patients in different disease maps, we can show that patients with some SNPs are found only in a small region of disease space, near the uninfected patients, suggesting these lucky individuals are resilient to malaria. We tested this idea using a relatively small existing dataset that provided little power. In the future, this sort of analysis could be used prospectively instead of retrospectively to design genetic studies that would differentiate resilient patients from studies that focus on severe pathogenesis.

The development of the concept of disease tolerance in animals provides hope that we can develop methods of reducing the impact of infections in ways that don’t depend upon the development of new antimicrobials. A frustration with this theory has been that the required parameters can only be measured in laboratory systems or, in rare exceptions, for chronic infectious diseases [14]. This means that we must argue by analogy when moving from our experimental systems to humans. Our approach of focusing on the progress of individuals as they loop through disease space overcomes this problem. This alternate method provides a second way of monitoring the output of an infection that works with cross-sectional data and provides the same sorts of insights we seek in studying disease tolerance.

## Methods

### Mice

C57Bl/6(J) female mice (8–12 wk) were obtained from Jackson Laboratory and maintained in the Stanford University animal facility. All experiments were performed in accordance with institutional guidelines and approved protocol (APLAC-26712). Mice were acclimated for 7–10 d prior to being used in all experiments.

### Temperature Probes

Mice were anesthetized locally with 1% lidocaine (4 mg/kg) and implanted subcutaneously with electronic temperature and ID transponders (IPTT-300 transponders, Bio Medic Data System, Inc) 1 wk prior to experimental *P*. *chaubaudi* infection. Temperatures were recorded every morning using a DAS-7006/7s reader (Bio Medic Data System, Inc).

### Infections

Infections were performed as described in [15,16] with some modifications. To limit variation, infections were started from an aliquot of the same parasite passage. Passage mice were infected intraperitoneally (i.p.) with 150 ul of thawed *P*. *chabaudi chabaudi* AJ (MR4/American type Culture Collection—MRA-756) infected red blood cells (iRBCs). Thin blood smears were prepared, Giemsa stained (Gibco-KaryoMAX), and counted daily until parasitemia reached 15%–20% (~7 d). Experimental mice were inoculated intraperitoneally with 100 ul containing 10^5^ iRBCs diluted in Krebs saline + glucose solution. Drinking water was supplemented with 0.05% 4-aminobenzoic acid to promote parasite growth.

### Infection Monitoring

All monitoring was performed in the morning between the hours of 9 AM and 12 PM to decrease variance due to circadian cycling. Weight and temperature were determined daily. Mice were restrained and their tails were warmed with sodium-acetate-based hand warmers to increase blood flow. The tail tips were nicked using sterilized surgical scissors, and approximately 18 ul of blood were collected daily for the course of the experiment. Blood was collected into an EDTA-coated 50 ul capillary tube and dispensed into EDTA-coated collection tubes to inhibit clotting. RBCs were counted as follows: 2 ul were pipetted into 1,000 ul of Hanks Balanced Salts Solution, identified using forward and side scatter, and counted using flow cytometery (Acuri C6). For parasite density measurement, 5 μl of blood were pipetted into 200 μl of sodium citrate saline-EDTA solution that was then centrifuged (1,000 x g, room temperature). The supernatant was removed and the pellets were stored at -80°C for later processing. For RNA extraction, 10 ul of blood was pipetted into 500 ul of RNA Later (Life Technologies) and stored at -80°C for later processing. The remaining 1 ul of blood was used to create a thin blood smear. These were Giemsa stained and examined under the microscope to count iRBCs to determine percent parasitemia.

### Determination of Parasite Density

Parasite density was calculated as the total number of infected RBCs (iRBCs) per unit volume of blood. This was calculated by multiplying the percentage of infected RBCs by the total number of RBCs per unit volume. This was done in two ways: by multiplying percent parasitemia by blood cell concentration and by qPCR to obtain a deeper dynamic range. Five ul of stored frozen blood was extracted using a DNA mini kit (Qiagen) according to the manufacturer’s instructions. *P*. *chabaudi* DNA was quantified from a standard curve via qPCR using primers for the Merozite Surface Protein 1 (MSP-1) (primers F- ACCAGCACAAGAAGCAACAA. R-TTGCGGGTTCTGTTGAGGCT) [17].

### Microarray

Two sets of microarrays were prepared. For microarrays for surviving mice, we prepared RNA from blood for three mice tracing similar patterns through phase space. We isolated RNA from days 0–25 for surviving mice and from days 0, 8, 10, 14, and 25 for control mice. For the microarrays for the mice who did not survive the infection, we processed samples from four mice that survived between 8 and 11 d post infection. Total blood RNA was isolated using a Mouse RiboPure Blood RNA isolation kit (Ambion-Life Technologies) following the manufacturer’s instructions. Purified RNA quantity and quality were measured at the Stanford Functional Genomics Facility using an Agilent 2100 BioAnalyzer and analyzed on the Illumina BeadArray Single Color platform (Illumina.Single.Color.MouseRef-8). Raw data was collected in Bead Studio and further processed in Genespring 12.1 GX.

### QRTPCR Analysis

RNA was isolated using the Mouse RiboPure-Blood RNA Isolation Kit (Life Technologies) and eluted in 40 ul of water. The RNA was converted into cDNA using the One-Step RT-PCR kit (Applied Biosystems), and the expression of ferrochelatase (Fech) was quantified using the following primers: F- TCATCCAGTGCTTTGCAGAC and R- CAGTGGCTCCTACCTCTTGG. A standard curve was created using RNA isolated from uninfected mice.

### Analysis

#### Mouse malaria data

Raw probe data from the Stanford Functional Genomics Facility was downloaded into Genespring 12.1 GX. Microarray samples were normalized using the 75% percentile normalization. Baseline transformation was applied using the median of all samples as the starting point. Genes were flagged “Present” above the cutoff of 0.8 and “Absent” below 0.6.

#### K-means analysis

All mouse gene probes (*n* = 25,697) were clustered into 20 groups using k-means analysis. The cluster number was determined using the k-means script in R to create a sum of squared error (SSE) scree plot to show the sum of squared errors associated with each cluster size. We chose a cluster size that provided the lowest variance with the smallest number of clusters. Clusters with an average 2-fold range of gene expression or higher were used for further analysis.

#### Nearest neighbor analysis

Nearest neighbor analysis was performed in MATLAB using the provided code available from the GitHub repository (https://github.com/bytorres/PlosBio2015). Data were analyzed using Euclidean distance as the metric.

#### Area analysis

The area of each phase plot was determined using MATLAB code available from the GitHub repository (https://github.com/bytorres/PlosBio2015). We assumed that the data points circumscribed a simple shape rather than crossed disease space, and the polyarea function was used to determine the area for each pairwise comparison of expression patterns. The 278 gene pairs that produced an area larger than an arbitrary cutoff of 11 were collected (S3 Table).

#### Human malaria data

Data (accession no. GSE34404) from Idaghour et al. [12] was loaded onto Genespring 12.1 GX and normalized as described above. Differences between infected and uninfected groups were analyzed using one-way analysis of variance (ANOVA) and loaded into Ayasdi Core.

#### Topological data analysis

Figs 3 and 4 were generated by performing topological data analysis (TDA) on the mouse and human datasets with the Ayasdi 3.0 software platform (ayasdi.com, Ayasdi Inc., Menlo Park, California). Nodes in the network represent clusters of infected mice and human patients, and edges connect nodes that contain samples in common. Nodes are colored by the average value of their samples for the variables listed in the figure legends. TDA was used to map the way hosts loop through the disease space in an unsupervised fashion. Mathematical details regarding the method of construction of the topological networks can be found in the following references: [13,18,19]. Two types of parameters are needed to generate a topological model: First is a measurement/notion of similarity, called a metric, which measures the distance between two points in some space (usually between rows in the data). Second are lenses, which are functions that describe the distribution of data in a space. A lens is a filter that converts a dataset into a vector, in which each row in the original dataset contributes to a real number in the vector; a lens operation turns every row into a single number. Metrics are used with lenses to construct the Ayasdi 3.0 output. Multiple lenses can be used in each analysis, and we used two types. When multiple lenses are used for an analysis, Ayasdi handles them mathematically by considering the Cartesian product. The first were lenses based on dimension reduction algorithms such as multidimensional scaling and nearest neighbor analyses, which helped analyze the data in an unsupervised manner. The second are lenses based on the data alone, for example, the levels of selectin expression or parasite density. We found that selectin expression was a marker that differed between living and dying mice when we used unsupervised metrics alone. When we added selectin (a cell adhesion molecule found in T cells) as a lens to the analysis, it helped separate the living and dying mice further than the unsupervised lenses on their own. The gene expression markers helped us dissect the graphs using knowledge about the biology of the system. In making a TDA graph, the points in the dataset are clustered within bins, defined by setting the resolution of the analysis. Resolution controls the number of bin partitions that will be created within the range of selected lens values; clustering then takes place within these bins. Increasing the resolution of a graph increases the number of bins. Bins sharing samples are connected by an edge. Gain defines the amount of oversampling between bins, and higher gain results in more edges. The metrics, lenses, resolution, and gain used in this paper are reported (See S6 and S9 Tables). Note that in the mouse studies, data from day 15 was censored to provide a cleaner graph. The infected mice typically suffer a recrudescence starting at this time, and this causes the looping curve to form cross bridges if this point is not removed. To determine how two groups of points defined in a TDA graph differ, a nonparametric statistical test (Kolmogorov-Smirnov) in combination with the *p*-value (*t* test) was used to identify parameters that had *p* < 0.05 for either one of the tests. Genes that were found to be expressed at significantly different levels are reported in S9 Table.

#### Polar transformation and statistical analysis

Data was transformed from Cartesian to polar coordinates using MATLAB code and analyzed using R code, both available from the GitHub repository (https://github.com/bytorres/PlosBio2015). The MATLAB code finds the center of a two-dimensional point cloud by first normalizing the ranges of the two dimensions. The center is identified by testing possible centers within the range of the data and identifying the one producing the smallest variance for calculated radii. We defined 0 degrees as day 0 of the infection. The R code is used to perform ANOVA over selected ranges of angles as well as to perform logistic regression analysis.

#### Human data analysis

For the experiment reported in Fig 8, the human data were further processed, and six samples were removed due to genomic kinship (identical by state ≥ 0.95) using the ProbABEL-package from the GenABEL suite of programs in R [20] on the data from [12].

## Supporting Information

S1 Data Data for Fig 5. (XLSX)

S2 Data Data for Fig 6. (XLSX)

S3 Data Data for Fig 7. (XLSX)

S4 Data Data for Fig 8. (XLSX)

S1 Fig Identification of k-means groups. We characterized k-means groups based on the overrepresentation of cell-type specific genes from the immunological genome project database [10]. In the figure, we show the overrepresentation of markers for each group and the timelines for the average gene expression values of each selected k-means group. Hypergeometric means and *p*-values supporting this analysis are reported in S2 Table.
(EPS)

S2 Fig Non-averaged disease space maps for malaria-infected mice. (A) The paths for eight parameters for three mice measured daily for 20 d are plotted in a timeline. The transcript markers used to define B cells, NK cells, granulocytes, and reticulocytes are, respectively, Cd79b, Nkg7, Camp, and Trim 10, and are reported as log_2_ values. Time is indicated by the increasing thickness of the curve.
(EPS)

S3 Fig Theoretical analysis of disease maps. (A) Randomly distributed points across a circle, with a similar range as the RBC by Fech space in (B), to represent a dense and complete loop. Each point represents an individual. (B) The sparse and asymmetrical distributed data found in the Idagdhour study [12] for the RBC by Fech space. (C) 95% confidence intervals across a series of sample group sizes for (A) expected (red) and (B) observed (blue) data. Having samples across the space provides tighter 95% confidence intervals.
(EPS)

S1 Table K-means groups for microarray analysis. This is the entire mouse gene set and is unfiltered for fold change or significance. K-means groups, gene symbol, reference number, Probes, and gene definitions are provided (referring to Fig 2).
(XLS)

S2 Table Cell type-specific transcript lists. Cell type-specific transcript list from references [10,11] used to select mouse genes listed in S1 Table.
(XLS)

S3 Table Classifying clusters to specific cell-type signatures. Hypergeometric means for cell type identification in S1 Fig.
(XLS)

S4 Table Genes producing large area loops. The gene pairs producing looping areas over 11 are listed (referring to Fig 2) (See Methods for available code).
(XLSX)

S5 Table Data used for nearest neighbor analysis. Probes used for mouse transcriptome nearest neighbor analysis shown in Fig 3. These are the 2-fold modulated genes that overlapped with immgen.org cell-specific groups [10] or reticulocyte-specific genes [11] (See S2 Table and available code in Methods).
(XLSX)

S6 Table TDA settings and color scale. Settings used for Ayasdi Core TDA analyses and color ranges used for Fig 3.
(XLSX)

S7 Table Data used for TDA. Live and dead mouse transcriptome data used for Fig 4.
(XLSX)

S8 Table TDA settings and color scale. Settings used for Ayasdi Core TDA analyses and color ranges used for Fig 4.
(XLSX)

S9 Table TDA statistical results for differences in live versus dead mice. Statistical table identifying transcripts differing between live and dead mice in Fig 4.
(XLSX)

S10 Table qRT-PCR data used to apply statistical analysis on disease maps. qRT-PCR data for live and dead mice used to do polar transformation and statistical analysis in Figs 5 and 7 (see Methods for available code).
(XLSX)

## Abbreviations

AS: Hemoglobin S
Fech: ferrochetalase
GWAS: genome-wide association study
NK: natural killer
qRT-PCR: quantitative reverse transcription polymerase chain reaction
RBC: red blood cell
TDA: topological data analysis
